# Effects of Social Defeat Stress on Sleep in Mice

**DOI:** 10.3389/fnbeh.2017.00227

**Published:** 2017-11-28

**Authors:** Fiona Henderson, Vincent Vialou, Salah El Mestikawy, Véronique Fabre

**Affiliations:** ^1^INSERM, CNRS, Neurosciences Paris Seine - Institut de Biologie Paris Seine (NPS - IBPS), Sorbonne Universités, UPMC Univ Paris 06, Paris, France; ^2^Department of Psychiatry, Douglas Mental Health University Institute, McGill University, Verdun, QC, Canada

**Keywords:** stress, social defeat, susceptibility, animal model, sleep, arousal, EEG activity, insomnia

## Abstract

Stress plays a key role in the development of psychiatric disorders and has a negative impact on sleep integrity. In mice, chronic social defeat stress (CSDS) is an ethologically valid model of stress-related disorders but little is known about its effects on sleep regulation. Here, we investigated the immediate and long-term effects of 10 consecutive days of social defeat (SD) on vigilance states in C57Bl/6J male mice. Social behavior was assessed to identify susceptible mice, i.e., mice that develop long-lasting social avoidance, and unsusceptible mice. Sleep-wake stages in mice of both groups were analyzed by means of polysomnographic recordings at baseline, after the first, third, and tenth stress sessions and on the 5th recovery day (R5) following the 10-day CSDS. In susceptible mice, each SD session produced biphasic changes in sleep-wake states that were preserved all along 10-day CSDS. These sessions elicited a short-term enhancement of wake time while rapid eye-movement (REM) sleep was strongly inhibited. Concomitantly, delta power was increased during non REM (NREM) sleep. During the following dark period, an increase in total sleep time, as well as wake fragmentation, were observed after each analyzed SD session. Similar changes were observed in unsusceptible mice. At R5, elevated high-frequency EEG activity, as observed in insomniacs, emerged during NREM sleep in both susceptible and unsusceptible groups suggesting that CSDS impaired sleep quality. Furthermore, susceptible but not unsusceptible mice displayed stress-anticipatory arousal during recovery, a common feature of anxiety disorders. Altogether, our findings show that CSDS has profound impacts on vigilance states and further support that sleep is tightly regulated by exposure to stressful events. They also revealed that susceptibility to chronic psychological stress is associated with heightened arousal, a physiological feature of stress vulnerability.

## Introduction

Mental disorders are a health priority as they are among the major contributors to the global burden of disease due to their high prevalence and high disability (Whiteford et al., 2013; Trautmann et al., 2016). Depressive and anxiety disorders carry the heaviest burden representing ~60% of disability-adjusted life years caused by mental and substance use disorders (Whiteford et al., 2013). The World Health Organization predicts depression as the greatest cause of disability worldwide by 2030, and the economic costs of mental disorders are expected to double. Therefore, there is an urgent need for a better characterization of the cardinal features of mental disorders. Sleep disturbances are a core symptom of psychiatric disorders as set out in the current diagnostic manual DSM-V (www.dsm5.org). Insomnia, defined as a complaint of difficulty initiating or maintaining sleep, is present in over 80% of patients with depression (Armitage, 2007). Furthermore, 40% of patients with insomnia have a coexisting psychiatric condition. Objective measurement of sleep using electroencephalogram (EEG) recordings found characteristic sleep disturbances in patients with depression and anxiety disorders including prolonged sleep latency, increased nocturnal awakenings and early morning awakenings (for reviews see Reynolds and Kupfer, 1987; Benca et al., 1997; Armitage, 2007; Steiger and Kimura, 2010). Sleep efficiency and continuity are also impaired as evidenced by decreased deep slow-wave sleep and slow-wave (delta) activity. Changes in rapid eye-movement (REM) sleep are described in depressed patients with reduced REM sleep latency, prolonged first REM sleep episode and elevated REM density (number of REM per REM sleep time). Similarly, studies on patients with post-traumatic stress disorder (PTSD) report an increase in REM density (Ross et al., 1989, 1994; Mellman et al., 1995; Pillar et al., 2000; Kobayashi et al., 2007; Germain, 2013). However, taken altogether, polysomnographic studies conducted in patient with depression and PTSD have yielded contradictory results, in particular for REM sleep, highlighting the complexity of using sleep biomarkers in these diseases (as described in the following reviews: Pillar et al., 2000; Buysse et al., 2006; Armitage, 2007; Germain, 2013 and for an overview see the following meta-analysis reports: Benca et al., 1992; Kobayashi et al., 2007; Pillai et al., 2011; Baglioni et al., 2016). These conflicting findings may be due to confounding factors, analysis done at different stages of the illness, disease heterogeneity, and comorbidities with other psychiatric conditions. Translational studies in animal models might be useful to define the exact relationship between sleep and affective disorders. For decades, extensive work in preclinical research has generated a large array of animal models sharing relevant endophenotypes of mental disorders. Although much work has been done on depressive- and anxiety-like behaviors, sleep profiling in animal models remains poorly explored in spite of being highly translational. Indeed, polysomnographic and quantitative EEG, the objective gold standard methods for sleep assessment, are carried out and analyzed with the same methods in both humans and animals.

In the recent years, social-stress based models have provided mechanistic insights on how stress shapes socioaffective behaviors. These models are believed to be relevant to the human situation as exposure to stressful life events is a major contributing factor to psychiatric disorders including major depression and anxiety disorders (Yehuda et al., 1998; Liu and Alloy, 2010). In particular, social stressors including social exclusion, low social status, bullying in workplaces or unemployment, have severe consequences on mental health (Björkqvist, 2001). Interestingly, exposure to social stress is translatable to rodents as social hierarchy and confrontation between conspecifics occurs naturally in males. Some studies suggest that chronic social stressors such as, an instable social environment are also applicable to females (Schmidt et al., 2010). Based on brief social subordination sessions in males, the chronic social defeat stress (CSDS) is an ethologically, face, and predictive valid approach for modeling stress-related disorders such as, depression, generalized anxiety, and/or PTSD (Franklin et al., 2012; Slattery and Cryan, 2014). CSDS is a well-studied model that induces long-term physiological and behavioral changes (Hammels et al., 2015). Thus, defeated mice develop a wide range of depressive-like behaviors including anhedonia but also social avoidance that can be normalized by chronic treatment with antidepressants (Berton et al., 2006). They also display anxiety-like behaviors (Berton et al., 2006; Krishnan et al., 2007). Physiological changes in defeated mice include decreased body weight, circadian abnormalities, or sensitized corticosterone reactivity (Krishnan et al., 2007). Some of these features, such as anhedonia, return shortly to normal levels after the stress paradigm. However, others including social avoidance, anxiety-like behavior or social hyperthermia persist up to 4 weeks. Although the majority of defeated mice express these passive coping responses and are considered as susceptible, some show stress resilience manifested by resistance to defeat-induced social avoidance (Krishnan et al., 2007). The mechanisms underlying stress resilience and susceptibility have been proposed to rely on the recruitment of specific neural circuits and neurochemical systems (Tornatzky and Miczek, 1993; Koolhaas et al., 1997; Berton et al., 2006; Krishnan et al., 2007; Vialou et al., 2014, 2015).

Although sleep impairments are important features of stress-related disorders, there are surprisingly few studies on the consequences of CSDS on sleep in mice (Olini et al., 2017; Wells et al., 2017). In the present study, we evaluated the impact of CSDS on sleep-wake stages and sleep EEG in susceptible mice. For this purpose, adult male mice of the C57Bl/6J background were subjected to 10-day CSDS and susceptible mice were subsequently identified in the social interaction test. The effects of social defeat on sleep/wake states were analyzed at different time-points throughout the CSDS paradigm and during the recovery period in susceptible and non-susceptible mice. Our findings reveal that CSDS markedly disturbs sleep and triggers long-lasting EEG changes.

## Materials and methods

### Animals and housing

Adult male C57Bl/6J (13–19 week-old, bodyweight: 23–30 g) and outbred male Swiss CD1 mice (9–16 week-old, body weight: 30–35 g) were used in this study (Centre d'élevage R. Janvier, Le Genest St. Isle, France). Mice were housed up to five per cage under standard conditions (12 h light/dark cycle; lights on at 7:00 a.m.; 22 ± 2°C ambient temperature; 60% relative humidity; food and water *ad libitum*). All experiments were performed in strict conformity with the European Union laws and policies for use of animals in neuroscience research (European Committee Council Directive 2010/63/EU) and were authorized by the Ethical Committee for Preclinical Research (Comité d'éthique en expérimentation animale Charles Darwin, CE2A-nb 5) of the French Ministry of Research and High Education (articles R.214-124, R.214-125).

### Surgery and electrode implantations

Mice were anaesthetized with ketamine/xylazine [50 and 2 mg/kg, respectively, intraperitoneal (i.p.) injection] before being fixed on a stereotaxic apparatus. All coordinates are adapted from the mouse brain atlas (Franklin and Paxinos, 2008). Mice were implanted with classical set of electrodes (made of enameled nichrome wire, 150 μm in diameter) for polysomnographic sleep monitoring (Boutrel et al., 1999). Briefly, two EEG electrodes were placed onto the dura through holes perforated into the skull over the right parietal cortex (2 mm lateral to midline and 2 mm caudal to bregma suture) and the cerebellum (at midline, 2 mm posterior to lambda), two electrooculogram (EOG) electrodes were located subcutaneously on each side of the orbit and two electromyogram (EMG) electrodes were inserted into the neck muscles. All electrodes were soldered to a miniconnector (Antelec, La Queue en Brie, France). Dental acrylic was used to anchor the electrodes to the skull. Mice were allowed to recover for 10 days and briefly handled daily for habituation. They were acclimated to the recording conditions for 4 days before starting the recordings and the behavioral experiments. Thus, mice were placed in individual recording chambers [custom-made Plexiglas cages (19 × 19 × 30 cm)] and connected to the recording system with a light-weight cable and a swivel allowing free movements.

### Sleep recordings and analysis

#### Recordings

**Figure 1:**
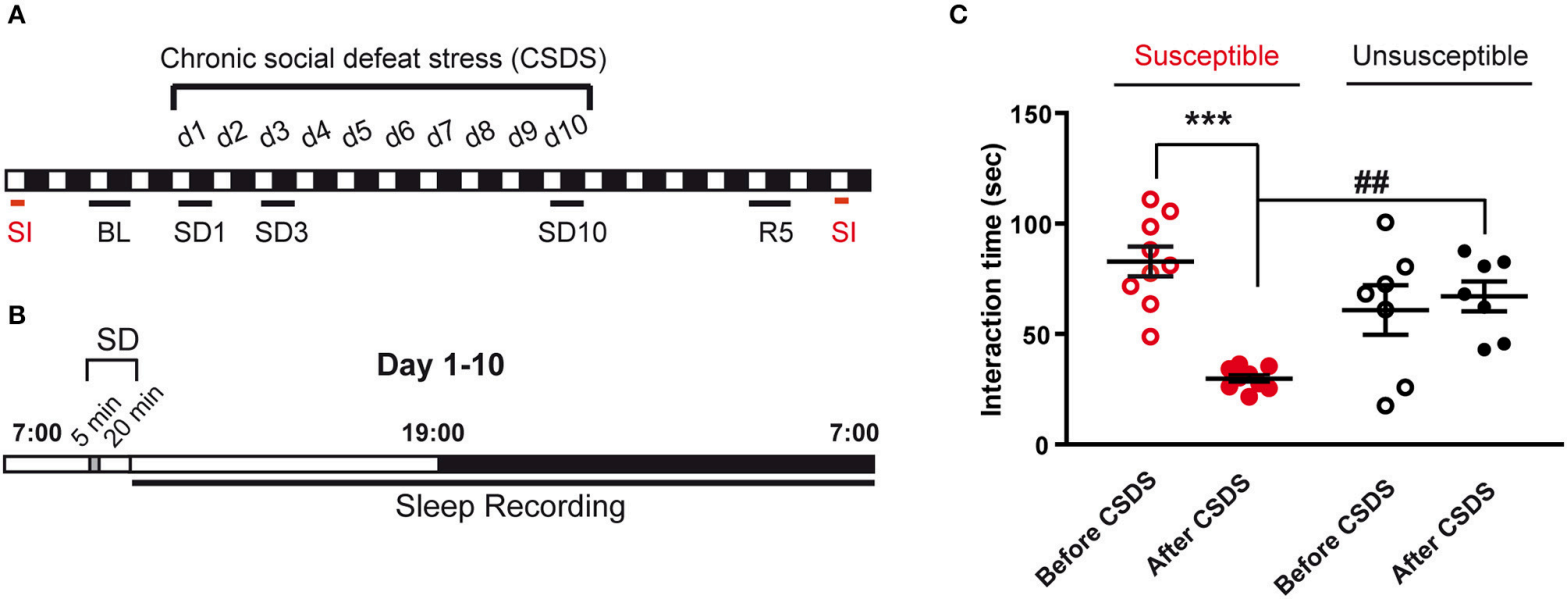
Experimental timeline and behavioral consequences of the chronic social defeat stress protocol. **(A)** Mice were exposed to chronic social defeat stress (CSDS) for 10 days (d1–d10). Analyzed polysomnographic recordings are indicated by black bars above the respective recording days (BL, SD1, SD3, SD10). As control, baseline (BL) recordings started 2 days before CSDS (24 h starting at 7 a.m.). Recordings were also collected on recovery day 5 (R5; 24 h starting at 7 a.m.). Mice were assessed for social avoidance behavior in the social interaction test (SI, red bars) before and after 10-day CSDS. **(B)** The CSDS paradigm consists in exposing mice to 5 min of physical defeat at 9:30 a.m. followed by 20 min of protected sensory contact with a trained CD1 aggressor male mouse. Mice were then placed back in their home cage and ploysomnographic recordings were started until the following day (7 a.m.). **(C)** Time in the interaction zone before (open circles) and after (filled circles) the 10-day CSDS paradigm. Mice were first allowed to explore for 2.5 min an arena in which an empty perforated plexiglass cage was placed on the middle of one wall. Then, an unfamiliar CD-1 mouse was introduced into the empty cage for 2.5 min (“Target” condition). Susceptible mice (red, *n* = 9) were selected *a posteriori* as follow: (1) a social interaction ratio [(interaction time, condition “Target” after the CSDS)/(interaction time, condition “Target” before the CSDS)] smaller than 1 and (2) an interaction time with target <45 s. Mice that did not reach these criteria were included in the unsusceptible group (black, *n* = 7). Data are expressed as means ± SEM of nine susceptible mice and seven unsusceptible mice. ^***^P < 0.001, significantly different from the control condition (before the CSDS paradigm). ^##^*P* < 0.01, susceptible vs. unsusceptible mice (for statistics see Table S1).

The timeline for sleep recordings is depicted in Figures 1A,B. A 24 h baseline (BL) recording starting at light onset (7 a.m.) was done 2 days before the CSDS protocol. During the 10-day CSDS protocol, mice were recorded immediately after the social defeat (SD) session starting at 09:30 a.m. until the next day (7 a.m.). Mice were gently restrained for disconnection/connection to the recording system before and after the SD session. A 24 h recording starting at light onset (7 a.m.) was also done on the 5th recovery day (R5). Recordings were analyzed on BL, on stress sessions SD1, SD3, and SD10 and on R5.

#### Scoring

The polysomnographic signals were recorded and digitized with an Embla Module (Medcare, Reykjavik, Iceland). They were sampled at a rate of 200 Hz (EEG) or 100 Hz (EOG and EMG). The EEG signals were analog band-pass filtered at 0.5 and 90 Hz for low- and high-pass filters, respectively, with the presence of a powerline notch filter. EOG and EMG filters were set at 0.5 and 45 Hz for low- and high-pass filters, respectively. Using the Somnologica®software (Medcare, Reykjavik, Iceland), polysomnographic recordings were visually scored every 10 s epoch as wakefulness (W), non REM (NREM) sleep, or REM sleep following classical criteria as previously described (Boutrel et al., 1999).

#### Sleep parameters

Vigilance states amounts for each animal were expressed as minutes per 3 or 12 h intervals. The sleep architecture was assessed by calculating the mean duration and frequency of vigilance states bouts (a bout could be as short as one epoch).

#### Power spectra analysis

The EEG (parietal-cerebellum) signal was processed for power spectra analysis on the followings days: BL, SD1, SD3, SD10, and R5. On the basis of visual and spectral analysis, 10-s epochs with artifacts were visually identified and omitted from the spectral analysis. One susceptible mouse showed EEG recordings with wake artifact during more than 20% of the time and was excluded from the spectral analysis. Then, consecutive 10-s epochs were subjected to a fast Fourier transformation routine (FFT), yielding power spectra between 0.4 and 50 Hz with a 0.4 Hz frequency bins (Alexandre et al., 2008). For each animal and vigilance state, a spectrogram was obtained and the values of the power spectra were divided into 5 frequency bands: delta (0.5–4.99 Hz), theta (5–9.99 Hz), alpha (10–12.99 Hz), beta (13–29.99 Hz), and low gamma (30–50 Hz). Mean EEG power spectrum was obtained by averaging the power spectra of all 10-s epochs of a given state in 3 h or 12 h intervals. EEG power spectra for each of the five frequency bands were expressed as a ratio of the mean EEG power over all frequency bands (relative values) and were compared to BL.

### Behavioral tests

#### Chronic social defeat stress paradigm

We first selected aggressive CD1 mice according to published methods (Berton et al., 2006). Upon arrival, CD1 male mice were singly housed to habituate for a week. A C57BL/6J male was introduced into each agressors' cage. Mice were allowed to interact until the first fight occurs or 2 min. Immediately after the first attack mice were separated and latency to first fight was recorded. This was repeated for the next 2 days (3-day screening). At the end of the 3rd day the most aggressive CD1 male mice were kept in large cages (19 × 35 × 14 cm) for the rest of the experiment.

To allow sleep recordings in between SD sessions, we used a modified version of the CSDS paradigm as described in Challis et al. (2013). Experimental C57BL/6J mice were submitted to SD for 10 consecutive days on daily basis (Figure 1A). The mouse was exposed to an unfamiliar and aggressive male CD1 mouse for 5 min before being separated from the aggressor by placing a divider in the same cage for the next 20 min allowing sensory, but not physical, contact (Figure 1B). The SD session lasted 25 min starting at 09:30 a.m. The mouse was then placed back in its recording chamber and connected back to the recording cable until the following SD session.

#### Social interaction test

Five days before the CSDS paradigm and on recovery day 7 (R7), social avoidance behavior was evaluated in the two-trial social interaction test (Figure 1A). In the first trial (2.5 min), the experimental mouse explored a white Perspex arena (43 × 43 × 26 cm) that has an empty perforated plexiglass cage along one side (“No target” condition) in a 10 lux illuminated room. In the second trial (2.5 min), the experimental mouse was reintroduced into the arena with an unfamiliar CD1 aggressor placed in the plexiglass cage (“Target” condition). Between these two trials, the experimental mice were returned to their home cage for 1 min. The time spent interacting with the unfamiliar CD1 mouse was recorded by video tracking (Viewpoint). Susceptibility to the stress paradigm was defined by two criteria: a social interaction ratio [(interaction time, condition “Target” after the CSDS)/(interaction time, condition “Target” before the CSDS)] smaller than 1 and an interaction time with “Target” less than 45 s.

### Statistics

All data were analyzed using Prism 7.0 (GraphPad Software). Normality assumptions were first verified prior to the use of any parametric tests (D'Agostino & Pearson normality test). In case of violation of normality assumption, non-parametric tests were used. Statistical analyses are described in Table S1. For the social interaction test, two-way repeated measures (RM) ANOVAs were performed for stress (repeated measures, before and after CSDS) and susceptibility (susceptible vs. unsusceptible mice). The effects of CSDS on sleep and wake amounts, bout frequency and mean duration, as well as on EEG power spectrum data, were analyzed using one-way ANOVAs with repeated measure over the day of stress (BL, SD1, SD3, or SD10) for each analyzed time window (10–13 h, 13–16 h, or 12 h of the dark period). Two-way RM ANOVAs were also applied to vigilance state data during the recovery phase for stress (repeated measures, BL and R5) and time (repeated measures over 3-h segments). Paired *t*-test and non-parametric two-tailed paired Wilcoxon test were also employed to analyze EEG power bands during the light or the dark period on R5 compared to BL. When appropriate, ANOVAs were followed by Dunn's, Tukey's, or Sidak's *post-hoc* tests. Statistical significance was set at 0.05 for all procedures. All tests were performed on raw data.

## Results

### Identification of susceptible mice

On completion of the sleep recordings (R7), social behavior was evaluated in the social interaction test before and after the stress protocol to identify susceptible and unsusceptible mice (Figure 1). The corresponding statistical analysis is described in Table S1.

Over 17 mice submitted to CSDS, ~60% were considered susceptible according to the inclusion criteria indicated in the legend of Figure 1. Social behavior was greatly affected by the CSDS protocol in the susceptible group (Figure 1C). Time spent in the interaction zone with an unfamiliar CD-1 mouse was significantly decreased after CSDS (−64%, before vs. after CSDS; Figure 1C). In contrast, mice of the unsusceptible group did not display a significant decrease in interaction after the stress protocol (Figure 1C). We then analyzed the consequences of 10-day CSDS on vigilance states in susceptible and unsusceptible mice.

### SD induced sleep disturbances throughout the 10-day CSDS in susceptible mice

**Figure 2:**
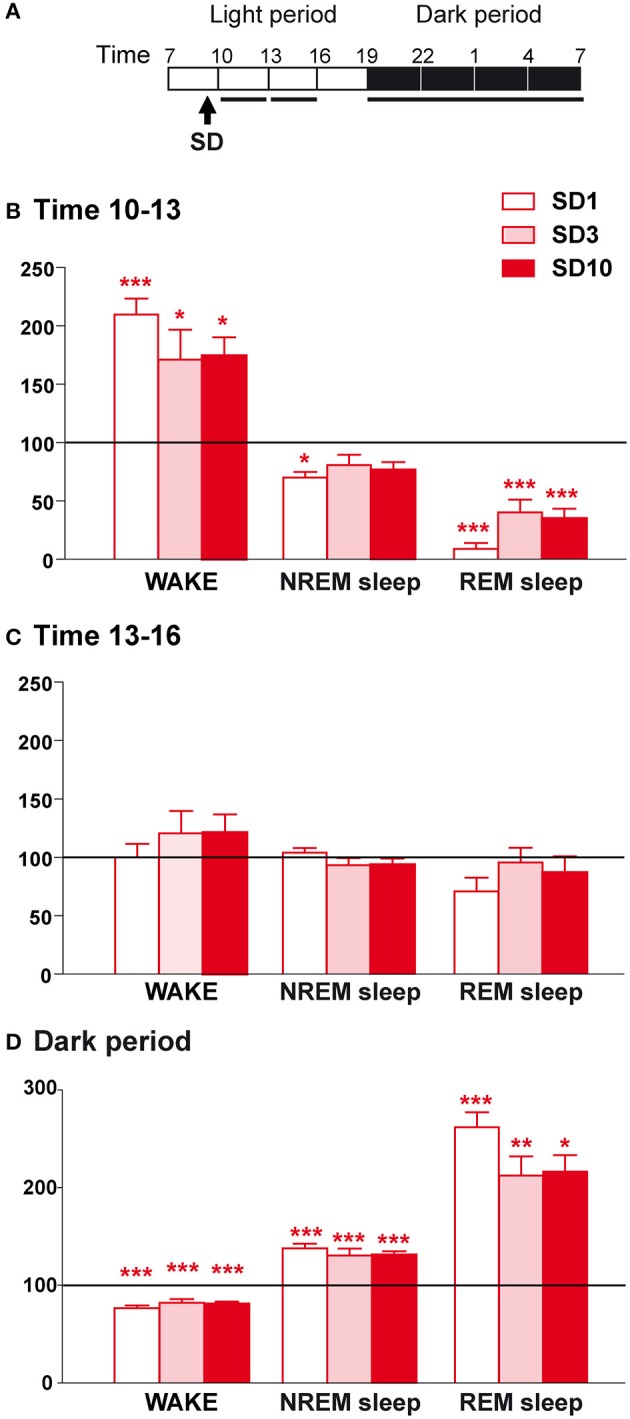
Sleep-wake changes during the first 6 h and the dark period after the social defeat session in susceptible mice. **(A)** Daily experimental timeline. Sleep-wake patterns were analyzed during the first two 3-h time windows and the 12 h of the dark period after the SD sessions as indicated by black bars above the respective recording days (SD1, SD3, SD10). **(B)** Amounts of wake, NREM sleep, and REM sleep expressed as minutes per 3 h for the time window 10–13 h on days SD1, SD3, and SD10 in susceptible mice (*n* = 9). **(C)** Amounts of wake, NREM sleep, and REM sleep expressed as minutes per 3 h for the time window 13–16 h on days SD1, SD3, and SD10 in susceptible mice (*n* = 9). **(D)** Amounts of wake, NREM sleep, and REM sleep expressed as minutes per 12 h of the dark period (7 p.m.−7 a.m.) on days SD1, SD3, and SD10 in susceptible mice (*n* = 9). Data, expressed as percentage of BL, are the means ± SEM of nine susceptible mice. Absolute values at BL (corresponding to 100%) were, in minutes per 3 h for the 2 time windows, respectively: 45 ± 4 and 46 ± 2 (wake), 124 ± 4 and 120 ± 2 (NREM sleep), and 13.6 ± 1.1 and 13.8 ± 1.4 (REM sleep). For the 12 h of the dark period, absolute values at BL (corresponding to 100%) were, in minutes per 12 h: 468 ± 14 (wake), 239 ± 12 (NREM sleep), and 13.4 ± 2.4 (REM sleep). Statistical analysis was performed on absolute values. For multiple group comparisons: ^*^P < 0.05, ^**^P < 0.01, and ^***^P < 0.001, significantly different from the control conditions (BL); for statistics see Table S1.

We evaluated the consequences of stress exposure on sleep-wake patterns during the course of 10-day CSDS. SD sessions were performed during the light “resting” period starting at 9:30 a.m. and vigilance states were analyzed on days SD1, SD3, and SD10 (Figure 2). The corresponding statistical analysis is described in Table S1.

When placed back in their recording chamber, susceptible mice showed a robust increase in wake amounts during the first 3 h period that follows the SD session relative to BL (Figures 2A,B). This wake enhancement reached significance for the different days tested indicating that the effects of SD on wake were maintained throughout the 10-day CSDS (Figure 2B). These changes were not due to modifications in the mean duration of wake bouts or their frequency (Table 1). Concomitantly, SD decreased NREM sleep amounts (Figure 2B). This stress effect was significant on SD1, but not on SD3 and SD10, despite a trend toward reduction. Nevertheless, during this period, REM sleep amounts were drastically reduced for all days tested (Figure 2B), resulting from a decrease in REM sleep bout numbers (Table 1). During the next 3 h period, wake and sleep amounts returned to normal levels compared to BL (Figure 2C). Thus, SD was found to promote arousal and inhibit REM sleep shortly after the SD session.

**Table 1:** Sleep and wake characteristics in susceptible mice during the first 6 h and the dark period after the SD session on days SD1, SD3, and SD10 of the CSDS.

		**Number of bouts**				**Bouts mean duration (min)**			
		**Baseline**	**SD1**	**SD3**	**SD10**	**Baseline**	**SD1**	**SD3**	**SD10**
WAKE	0–3 h	31 ± 3	29 ± 3	30 ± 5	23 ± 3	1.6 ± 0.3	3.5 ± 0.4	4.0 ± 1.4	4.3 ± 0.7
	3–6 h	26 ± 1	34 ± 3	30 ± 2	29 ± 3	1.8 ± 0.1	1.4 ± 0.2	1.9 ± 0.3	2.1 ± 0.3
	Dark	64 ± 4	99 ± 7^**^	87 ± 5^*^	92 ± 8^*^	7.7 ± 0.7	3.7 ± 0.3^**^	4.5 ± 0.5^**^	4.4 ± 0.5^*^
NREMS	0–3 h	31 ± 3	28 ± 3	30 ± 5	23 ± 3	4.1 ± 0.3	3.1 ± 0.3	3.7 ± 0.4	4.5 ± 0.5
	3–6 h	26 ± 1	34 ± 3	30 ± 2	29 ± 3	4.8 ± 0.3	3.9 ± 0.5	3.9 ± 0.5	4.2 ± 0.5
	Dark	62 ± 4	98 ± 7^**^	86 ± 5	91 ± 8^*^	3.9 ± 0.2	3.5 ± 0.3	3.7 ± 0.2	3.7 ± 0.3
REMS	0–3 h	11 ± 1	1 ± 1^***^	4 ± 1^*^	4 ± 1^*^	1.3 ± 0.1	1.1 ± 0.5	1.3 ± 0.1	1.1 ± 0.2
	3–6 h	10 ± 1	8 ± 1	9 ± 1	9 ± 1	1.4 ± 0.1	1.2 ± 0.1	1.4 ± 0.1	1.3 ± 0.1
	Dark	11 ± 2	25 ± 2^***^	22 ± 2^**^	22 ± 2^**^	1.4 ± 0.2	1.5 ± 0.1	1.3 ± 0.1	1.4 ± 0.1

The data are given as means ± S.E.M (n = 9). For multiple group comparisons:

* *P < 0.05*,

** P < 0.01, and

*** *P < 0.001 compared to BL. The statistical analysis is described in Table S1*.

In addition, SD elicited a sleep rebound at the expense of wake levels during the following dark “active” period (Figures 2A,D). Indeed, susceptible mice showed increased time spent in NREM sleep on days SD1, SD3, and SD10 (Figure 2D). This effect was the consequence of an increase in the number of NREM sleep bouts, whose mean duration remained unchanged (Table 1). In parallel, SD markedly enhanced REM sleep amounts throughout the 10 SD sessions reaching a +150% increase on SD1 relative to BL (Figure 2D). This enhancement was due to an increase in the number of REM sleep bouts, with no modification in their mean duration (Table 1). Concomitantly, susceptible mice showed a decrease in wake amounts during the “active” dark period that follows the SD sessions (Figure 2D). Interestingly, the nocturnal wake architecture was also impacted by stress. On SD1, the number of wake bouts increased by +55% whereas their mean duration decreased by −52%. This inability to maintain long wake bouts is indicative of wake instability. As for its diurnal consequences, nocturnal sleep changes (namely sleep rebound and wake instability) were maintained throughout the 10-day CSDS (Figure 2D).

Taken together, these results show that SD sessions induced a powerful disruption of sleep in susceptible mice including: (1) immediate marked arousal and a concomitant drop in REM sleep amounts during the “resting” light period and (2) delayed increase in sleep and wake instability lasting the entire following “active” dark period. These alterations of sleep-wake states were observed from the first to the tenth SD session indicating that stress-induced sleep changes were preserved throughout 10-day CSDS.

### SD induced EEG power spectrum changes during NREM sleep all along the 10-day CSDS in susceptible mice

**Figure 3:**
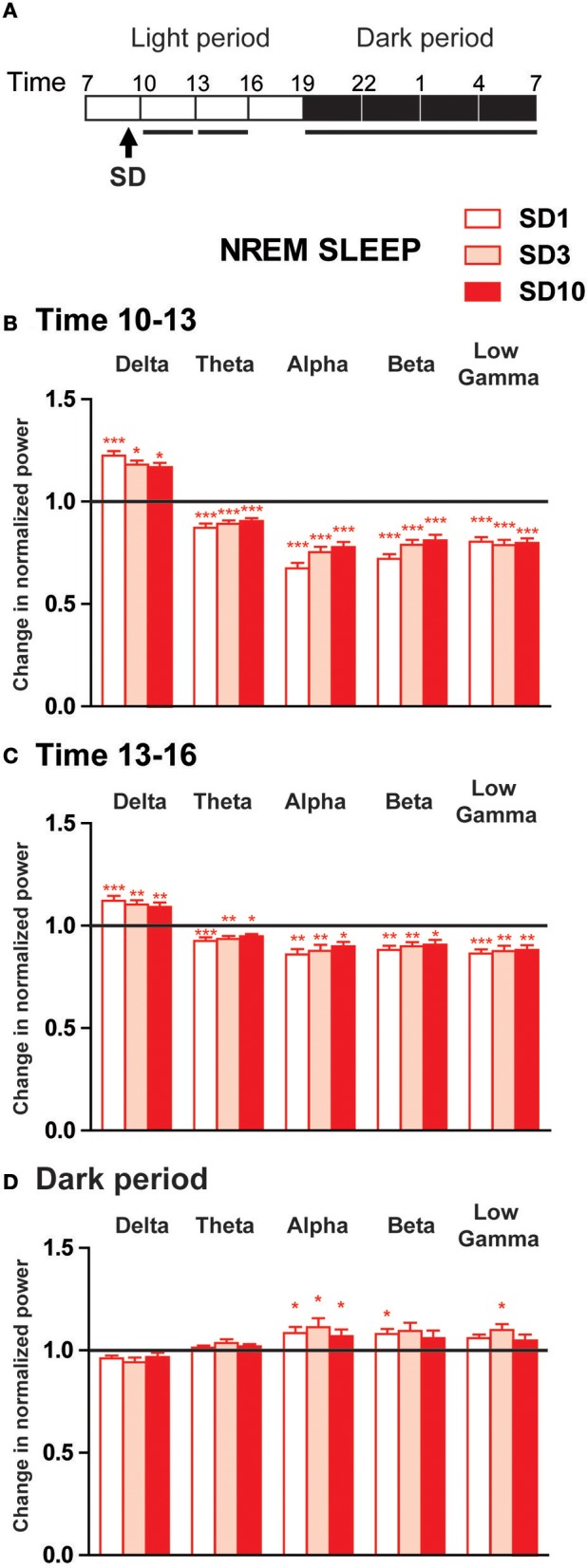
Power spectra analysis of the EEG frequency bands within NREM sleep during the first 6 h and the dark period after the social defeat session in susceptible mice. **(A)** Daily experimental timeline. Power spectra were analyzed during the first two 3-h time windows and the 12 h of the dark period after the SD sessions as indicated by the black bar above the respective recording days (SD1, SD3, SD10). **(B–D)** Changes in normalized relative EEG power spectrum on days SD1, SD3, and SD10 compared to BL during NREM sleep in susceptible mice (*n* = 8). For each time window tested during the light period [10–13 h **(B)** and 13–16 h **(C)**] and during the dark period [7 p.m.−7 a.m. **(D)**], changes in relative normalized EEG power in each power band (delta: 0.5–4.99 Hz, theta: 5–9.9 Hz, alpha: 10–12.99 Hz, beta: 13–29.99 Hz and low gamma: 30–50 Hz) were expressed as a ratio of individual relative values obtained on each SD session over BL. For each mouse, relative values were obtained by dividing each power band over the sum of all values from 0.5–50 Hz. All data are expressed as means ± SEM of eight susceptible mice. Statistical analysis was performed on relative EEG power in each power band. For multiple group comparisons: ^*^P < 0.05, ^**^P < 0.01, and ^***^P < 0.001, significantly different from the control conditions (BL); for statistics see Table S1.

Sleep quality, as revealed by elevated delta power during NREM sleep, depends on the duration of previous wake but also on its nature (Meerlo and Turek, 2001). We therefore analyzed EEG power density during NREM sleep on days SD1, SD3, and SD10 of the 10-day CSDS relative to BL (Figure 3 and see Table S1 for statistics).

During the first 3 h period after the SD session, susceptible mice showed increased EEG activity in the delta range (0.5–4.99 Hz; Figures 3A,B). Conversely, SD consistently elicited a significant decrease in theta to low gamma power during NREM sleep (Figure 3B). Similar changes of a lesser extent were observed during the next 3 h period (Figure 3C). These diurnal modifications were maintained throughout 10-day CSDS (Figures 3B,C). During the dark period, EEG power spectra in defeated mice showed a mirror image with enhanced fast frequencies during NREM sleep on days SD1, SD3, and SD10 (Figure 3D). Thus, alpha power was significantly enhanced on all days tested (Figure 3D). Increased EEG activity was also observed in the beta and low gamma bands but these differences were significant only on days SD1 and SD3, respectively (Figure 3D).

Altogether, these results show that SD produced biphasic changes in quantitative EEG power spectra during NREM sleep in susceptible mice. During the light phase, delta activity was enhanced after the SD sessions suggesting that stress-induced arousal first challenged the sleep homeostatic process. During the subsequent dark phase, defeated mice showed increased high-frequency EEG activity that was mainly observed in the alpha band. As described for sleep-wake states, SD-induced changes in EEG power spectra were, for the most part, maintained throughout the 10-day CSDS.

### SD induced sleep and EEG power spectrum changes throughout the 10-day CSDS in unsusceptible mice

**Figure 4:**
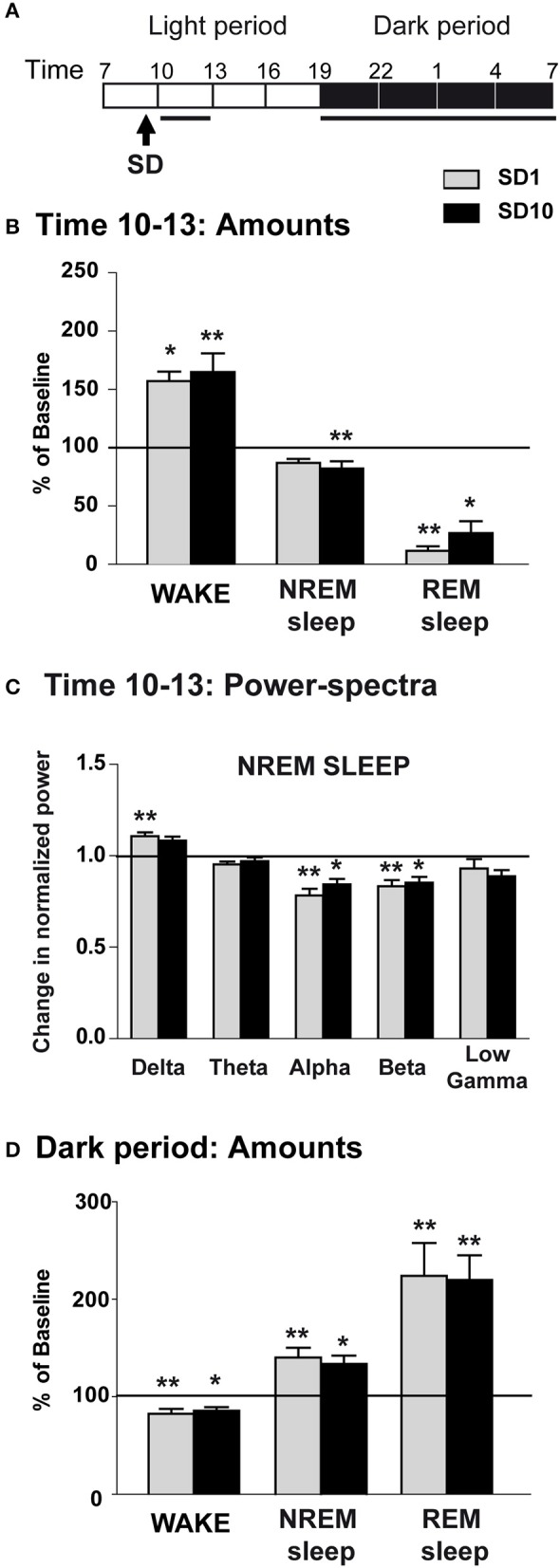
Sleep-wake changes during the first 3 h and the dark period after the social defeat session in unsusceptible mice. **(A)** Daily experimental timeline. Sleep-wake patterns were analyzed during the first 3-h time window and the 12 h of the dark period after the SD sessions. Power-spectra were analyzed during the first 3-h time window of the light period after the SD session. Analyzed time windows are indicated by black bars above the respective recording days (SD1 and SD10). **(B)** Amounts of wake, NREM sleep and REM sleep expressed as minutes per 3 h on days SD1 and SD10 in unsusceptible mice (*n* = 7). Sleep-wake states were analyzed for the first 3-h time window after the SD session (starting at 9:30 a.m.) and compared to results obtained from BL recordings. Data are expressed as percentage of BL and absolute values at BL (corresponding to 100%) were, in minutes per 3 h: 49 ± 4 (wake), 119 ± 4 (NREM sleep) and 11.9 ± 0.9 (REM sleep). **(C)** Changes in normalized EEG power spectrum on days SD1 and SD10 compared to BL during NREM sleep in unsusceptible mice (*n* = 7). Changes in normalized EEG power in each power band (delta: 0.5–4.99 Hz, theta: 5–9.9 Hz, alpha: 10–12.99 Hz, beta: 13–29.99 Hz, and low gamma: 30–50 Hz) were expressed as a ratio of individual relative values obtained on each SD session over BL. For each mouse, relative values were obtained by dividing each power band over the sum of all values from 0.5–50 Hz. **(D)** Amounts of wake, NREM sleep and REM sleep expressed as minutes per 12 h of the dark period on days SD1 and SD10 in unsusceptible mice (*n* = 7). Sleep-wake states were analyzed for the 12 h of the dark period following the SD session (starting at 9:30 a.m.) and compared to results obtained from BL recordings. Data are expressed as percentage of BL and absolute values at BL (corresponding to 100%) were, in minutes per 12 h of the dark period: 490 ± 12 (wake), 215 ± 10 (NREM sleep) and 14.6 ± 2.3 (REM sleep). All data are expressed as means ± SEM of seven unsusceptible mice. Statistical analysis was performed on absolute values. For multiple group comparisons: ^*^P < 0.05, ^**^P < 0.01, significantly different from the control conditions (BL); for statistics see Table S1.

The CSDS paradigm makes possible to examine variability in behavioral responses. Beside susceptible individuals, 10-day CSDS produces mice that fail to develop social avoidance, i.e., the unsusceptible mice. To test whether these mice show specific stress-induced sleep changes, we evaluated vigilance states and EEG power density in unsusceptible mice throughout the CSDS. As sleep changes were similar all along the 10-day protocol in susceptible mice, we restricted our analysis to days SD1 and SD10 (Figure 4 and Table 2). The corresponding statistical analysis is described in Table S1.

**Table 2:** Sleep and wake characteristics in unsusceptible mice during the first 3-h and the dark period after the SD session on days SD1 and SD10 of the CSDS.

		**Number of bouts**			**Bouts mean duration (min)**		
		**Baseline**	**SD1**	**SD10**	**Baseline**	**SD1**	**SD10**
WAKE	0–3 h	25 ± 2	30 ± 2	22 ± 4	2.1 ± 0.3	2.7 ± 0.2	4.8 ± 1.1
	Dark	54 ± 3	94 ± 6^**^	81 ± 5	9.4 ± 0.6	4.2 ± 0.6^*^	4.9 ± 0.4
NREMS	0–3 h	25 ± 2	30 ± 2	23 ± 4	5.0 ± 0.4	3.4 ± 0.3^*^	4.8 ± 0.6
	Dark	52 ± 3	93 ± 7^**^	80 ± 5	4.8 ± 0.3	3.4 ± 0.2	3.9 ± 0.3
REMS	0–3 h	10 ± 1	2 ± 1^***^	3 ± 1^*^	1.2 ± 0.1	0.7 ± 0.1^*^	1.0 ± 0.2
	Dark	14 ± 3	25 ± 4^**^	27 ± 3	1.1 ± 0.1	1.3 ± 0.1	1.2 ± 0.1

The data are given as means ± S.E.M (n = 7). For multiple group comparisons:

* *P < 0.05*,

** P < 0.01, and

*** *P < 0.001 compared to BL. The statistical analysis is described in Table S1*.

On days SD1 and SD10, unsusceptible mice displayed enhanced wake and decreased REM sleep amounts during the first 3 h following the SD sessions (Figures 4A,B). REM sleep reduction was accounted for by a decrease in the number of REM sleep bouts on all day tested and a decrease in their mean duration on SD1 (Table 2). Concomitantly, delta activity was significantly increased during NREM sleep on SD1 (Figure 4C). Despite a trend toward enhancement, delta activity remains statistically unchanged on SD10. Conversely, a significant decrease in alpha to beta power during NREM sleep was observed during this 3-h period on days SD1 and SD10 (Figure 4C). During the following dark period, SD elicited an increase in both NREM and REM sleep amounts for all day tested (Figure 4D). In addition to sleep enhancement, wake instability was observed on SD1 as shown by an increase in the number of wake bouts and a decrease in their mean duration (Table 2). Similar, but not significant, trends were observed on SD10 (Table 2).

Altogether, these data show that unsusceptible mice exhibit responses to SD that resemble those observed in susceptible mice including on a short-term basis: wake enhancement, reduced REM sleep and increased delta activity during NREM sleep. During the following dark period, SD promoted sleep and disrupted wake stability.

### Long-lasting sleep and EEG power spectrum changes after CSDS in susceptible and unsusceptible mice

We next investigated the effects of the CSDS paradigm on vigilance states on the 5th recovery day (R5) when susceptible mice have developed long-lasting social avoidance (Figure 1C).

**Figure 5:**
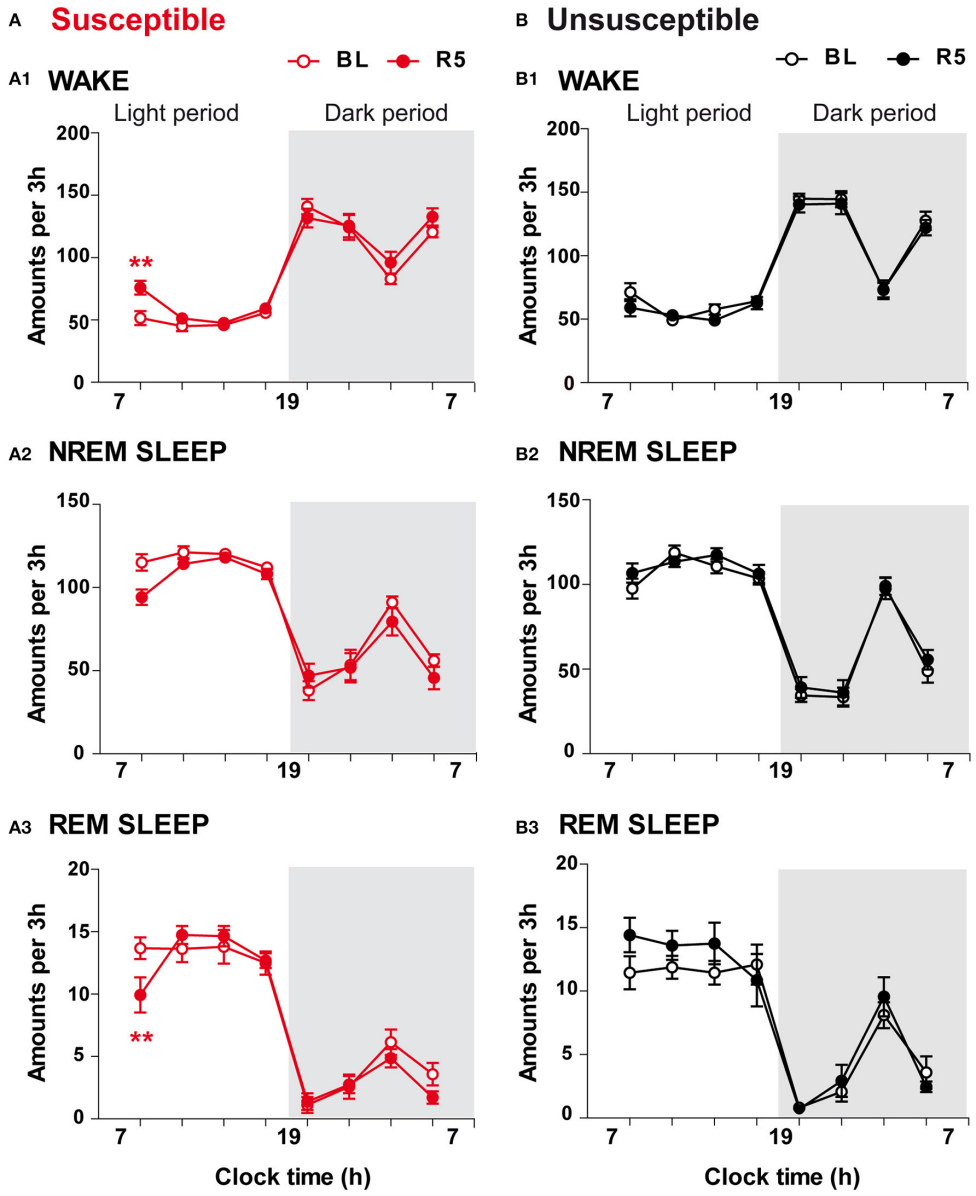
Long-term effects of the chronic social defeat stress on sleep-wake states in susceptible and unsusceptible mice. Amounts of vigilance states across the light/dark cycle in susceptible **(A)** and unsusceptible **(B)** mice. Amounts of wake **(A1,B1)**, NREM sleep **(A2,B2)**, and REM sleep **(A3,B3)** are expressed as minutes per 3 h across the light/dark cycle in susceptible mice (red symbols, *n* = 9) and in unsusceptible mice (black symbols, *n* = 7) at baseline (BL, open circles) and on recovery day 5 (R5, filled circles). All data, expressed in minutes per 3 h, are means ± SEM of nine susceptible and seven unsusceptible mice. ^**^P < 0.01, significantly different from the control conditions (BL); for statistics see Table S1.

On R5, CSDS had limited effects on sleep-wake patterns (Figure 5). Susceptible mice exhibited enhanced wake amounts (+47%; Figure 5A1) together with reduced REM sleep amounts (−27%; Figure 5A3) over the first 3 h of the light period (7–10 a.m.). Despite a trend toward reduction, NREM sleep amounts were not significantly affected during this period (Figure 5A2; for statistics, see Table S1). Analysis of the following 3-h time windows revealed that vigilance states after the CSDS paradigm were not significantly different from those recorded in BL conditions (Figure 5A). In contrast, 10-day CSDS did not affect vigilance states in unsusceptible mice. Thus, wake, NREM sleep and REM sleep amounts remained unchanged across the light/dark cycle on R5 compared with BL (Figures 5B1–B3).

**Figure 6:**
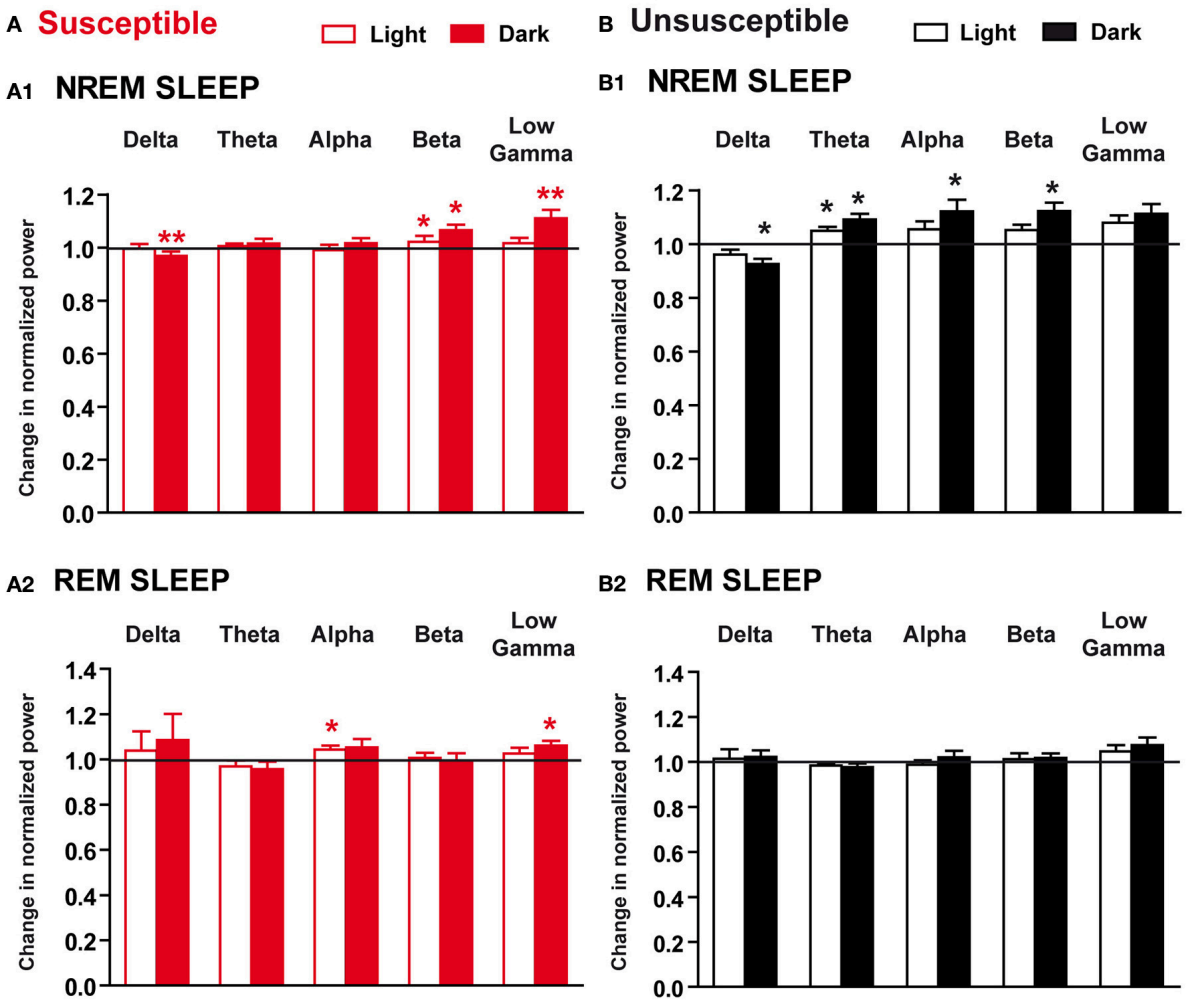
Long-term effects of the chronic social defeat stress on EEG power spectra within NREM sleep and REM sleep in susceptible and unsusceptible mice. Changes in normalized EEG power spectrum on recovery day 5 (R5) compared to BL in susceptible **(A)** and unsusceptible **(B)** mice during NREM sleep **(A1,B1)** and REM sleep **(A2,B2)**. For each time window tested (12-h of the light and 12-h of the dark periods), changes in normalized EEG power in each power band (delta: 0.5–4.99 Hz, theta: 5–9.9 Hz, alpha: 10–12.99 Hz, beta: 13–29.99 Hz, and low gamma: 30–50 Hz) were expressed as a ratio of individual relative values obtained on each SD session over BL. For each mouse, relative values were obtained by dividing each power band over the sum of all values from 0.5–50 Hz. All data are means ± SEM of eight susceptible and seven unsusceptible mice. Statistical analysis was performed on relative EEG power in each power band. ^*^P < 0.05, ^**^P < 0.01, significantly different from the control conditions (BL); Wilcoxon's or Student's paired *t*-tests (see Table S1).

To examine whether 10-day CSDS might disrupt sleep quality, we next performed EEG spectral analysis within NREM sleep and REM sleep in susceptible and unsusceptible mice. In susceptible mice, during NREM sleep, beta to low gamma activity was enhanced during the “active” dark period, while delta power was decreased on R5 (Figure 6A1). Similar trends were observed during the light period with a significant increase in beta rhythms during NREM sleep (Figure 6A1). Minor changes were observed during REM sleep with significant enhanced alpha and low gamma rhythms during the light and the dark phases, respectively (Figure 6B2). However, these changes were detected while the absolute EEG power was globally decreased in both NREM sleep (BL: 3.67 ± 0.24 and R5: 3.32 ± 0.30 in μV^2^; two-tailed paired *t*-test: *p* < 0.05) and REM sleep (BL: 2.12 ± 0.16 and R5: 2.00 ± 0.17 in μV^2^; two-tailed paired *t*-test: *p* < 0.05). In unsusceptible mice, long-term EEG power spectrum changes included reduced delta activity and enhanced theta to beta activity during NREM sleep (Figure 5B1). These changes were mainly observed during the dark “active” phase. Despite a trend toward enhancement, low gamma rhythms were not statistically different on R5 in unsusceptible mice (Figure 5B1). During REM sleep, EEG power spectrum remained unchanged on R5 compared to BL in unsusceptible mice (Figure 5B2). These modifications occur while the absolute EEG power was globally decreased by ~10% in both NREM sleep and REM sleep (two-tailed paired *t*-test; *p* < 0.05).

Altogether, our data show that the CSDS has discrete long-lasting consequences on sleep-wake patterns in susceptible mice including: (1) anticipatory arousal (enhanced wake) at the time corresponding to the SD session after cessation of the stress paradigm and (2) hallmarks of decreased sleep quality comprising high frequency activity during sleep. Unsusceptible mice showed no change in sleep-wake states but decreased sleep quality as evidenced by enhanced high frequency activity during NREM sleep on recovery.

## Discussion

The present study examined sleep-wake patterns and sleep architecture of mice exposed to CSDS. This model of stress-related disorders has a good face, construct, and pharmacological validity, reproducing in susceptible mice stress-induced social avoidance a behavioral trait commonly found in patients with depression, social phobia and PTSD. Our protocol was designed to analyze sleep-wake patterns and EEG power spectra throughout the 10-day CSDS and during recovery when susceptible mice show social avoidance. Exposure to acute SD stress during the sleep phase produced on defeated mice of both the susceptible and the unsusceptible subgroups a temporally dual effect on sleep that initially involved sustained wake and sleep loss with suppression of REM sleep. These changes were associated with an increase in sleep depth as revealed by higher low-frequency high-amplitude activity (delta power) during NREM sleep. During the following active phase, defeated mice displayed an increase in total sleep time. These overall sleep changes were maintained over the course of 10-day CSDS suggesting that sleep remains tightly regulated during repetition of stress. The majority of these alterations were normalized after cessation of the stress procedure. However, some sleep disturbances analogous to symptoms in patients with mood disorders emerged during recovery. Susceptible and unsusceptible mice display reduced delta power and augmentation of EEG high frequency activity during NREM sleep reminiscent of the EEG features found in insomniacs. Additionally, several days after cessation of the stress paradigm, we observed an anticipatory threat reaction characterized by an enhanced wake that was only present in susceptible mice. This anticipatory wake evokes the excessive anticipatory responses to future threat uncertainty commonly associated with anxiety.

### Preserved sleep-wake changes all along the chronic stress protocol

We observed a specific time course for sleep adaptations in response to acute SD stress that was maintained all along the 10-SD sessions of the CSDS. Acute SD stress first exerted a potent wake-enhancing action that lasted for several hours. This type of stress-induced arousal has been classically associated with activation of the autonomic and neuroendocrine systems that enables the organism to face threatening events (Dampney, 2015) and shown to involve wake promoting neurons, including hypocretin (España et al., 2003; Nollet et al., 2011), and norepinephrine (Ceccatelli et al., 1989) neurons. We found that acute SD stress also engaged the homeostatic regulation of sleep as shown by higher delta activity during NREM sleep which could be the consequence of sustained wake. This homeostatic regulation of sleep consists in a compensatory increase in sleep depth after prolonged wake reflected by delta activity, (Borbély, 1982, 2001; Achermann et al., 1993). Accordingly, delta activity peaks at sleep onset and is enhanced after sleep deprivation (Franken et al., 1991; Huber et al., 2000). Our result showing higher delta activity during NREM sleep after acute SD stress is consistent with findings in rats suggesting that homeostatic sleep regulation depends on both the duration and the intensity of prior wake (Meerlo and Turek, 2001; Kamphuis et al., 2015). In particular, sleep deprivation caused by an aggressive interaction had a more powerful impact on delta activity than the one induced by gentle handling (Meerlo and Turek, 2001). More importantly, we found that enhanced sleep depth after acute SD stress was maintained over the course of 10-day CSDS. This observation confirms that the homeostatic mechanisms of sleep regulation remain intact in the context of repeated challenges, as the case occurs during chronic sleep fragmentation (Baud et al., 2015) and chronic sleep restriction (Leemburg et al., 2010). This preserved regulation suggests that NREM sleep may play a fundamental role in the course of recovery, potentially by providing an optimal physiological state for clearing metabolites that have been accumulated during wake (Xie et al., 2013) and regulating extracellular ion homeostasis (Ding et al., 2016). Whether these restorative processes are engaged after the SD stress remains to be established.

In addition to NREM sleep alterations, our results show specific REM sleep changes throughout 10-day CSDS. First, wake enhancement after acute SD stress mainly occurred at the expense of REM sleep amounts. Following initial wake enhancement, there was a sleep rebound during the active period characterized by a large increase in REM sleep. Analogous adaptive sleep regulation has been reported in response to a large variety of stressors (for reviews see Pawlyk et al., 2008; Suchecki et al., 2012). Confrontation with an aggressive conspecific in rats (Meerlo and Turek, 2001), acute restraint stress in mice (Meerlo et al., 2001; Rachalski et al., 2009) and rats (Rampin et al., 1991), footshocks (Pawlyk et al., 2005), brief presentation of ether (Bodosi et al., 2000), or combination of these stressors (Nedelcovych et al., 2015) elicited a similar biphasic sleep response. As found for NREM sleep, these stress-induced REM sleep changes were maintained all along CSDS, indicating the preservation of the adaptive response in spite of stress repetition. A recent study also reported enhanced REM sleep amounts throughout the CSDS paradigm in mice (Wells et al., 2017). However, in contrast with our findings, the REM sleep rebound appeared progressively (Wells et al., 2017). This discrepancy might be due to longer daily bouts of SD (10 vs. 5 min in the present study) and longer protected sensory contact with the aggressor (continuous vs. 20 min in the present study) than in ours. Indeed, the length of the stressor appears to determine the magnitude of the REM sleep rebound, with longer stress inducing smaller REM sleep rebound (Marinesco et al., 1999). Another difference with the study of Wells et al. (2017) is that our protocol is sufficient to disrupt social behavior (see results) as previously shown by others (Challis et al., 2013, 2014). As for NREM sleep, we hypothesize that recurrent REM sleep rebound is part of the processes helping the organism to cope with stress. REM sleep has been proposed to play a fundamental role in the adaptive mechanisms induced by aversive experiences. It is believed to promote emotional memory consolidation and to weaken the emotional content related to a traumatic event (“Sleep to forget and sleep to remember” model, Goldstein and Walker, 2014). Thus, dysregulation of this emotional memory processing could be a core mechanism responsible for PTSD. In line with this model, victims of traumatic events who exhibit more consolidated REM sleep episodes after the trauma do not develop PTSD (Mellman et al., 2007). Further studies should establish the potential role of stress-induced REM sleep rebound in minimizing the negative impact of CSDS.

Overall, our results provide support for the tight relationship between sleep and stress. Importantly, acute SD-induced sleep changes are independent of the future stress outcomes, i.e., the social aversion, as susceptible and unsusceptible mice show similar sleep changes across the 10-day CSDS. Hence, sleep alterations during the CSDS protocol are not predictive of stress vulnerability but may rather help the organism to cope with stress. We thus propose that both NREM and REM sleep underlie restorative processes to face aversive events.

### Long-lasting sleep changes reminiscent of stress-related disorders

Initial enhancement of slow wave (delta) activity during NREM sleep and secondary sleep rebound can be considered markers of the ongoing recovery processes triggered by SD stress. We found, in contrast, sleep alterations during recovery that might underlie the long-lasting deleterious effects of chronic stress. Notably, we show that both susceptible and unsusceptible mice display reduced delta activity and enhanced power in higher frequency bands during recovery, a phenotype that emerged after the 10-day CSDS. This altered delta activity during NREM sleep is in line with a recent study showing disrupted sleep homeostasis in response to sleep deprivation in chronically defeated mice (Olini et al., 2017). Collectively, these data position delta activity as a central player in stress responses. Importantly, these EEG features during NREM sleep are reminiscent of primary insomnia. Indeed, EEG spectral characteristics in chronic insomniacs show augmented high-frequency EEG activity (Freedman, 1986; Merica et al., 1998; Perlis et al., 2001; Riedner et al., 2016). Increased power of rapid EEG bands is characteristic of attentional and cognitive processing engaged during wake, and, consequently, their presence during NREM sleep is a sign of persistent activity of the wake systems confirmed by neuroimaging studies in insomniacs (Nofzinger et al., 2004). This heightened wake-like EEG activation is believed to reflect hyperarousal of the brain during sleep and represent the core symptom of chronic insomnia. Interestingly, unsusceptible mice show additional increase in EEG activity in the theta and alpha bands during NREM sleep. A clinical study has reported that patients with primary insomnia exhibit more theta and alpha power during NREM sleep than those with major depression (Perlis et al., 2001). Together, these findings suggest that differences in the EEG activity can help discriminate individuals in light of their stress susceptibility. In summary, our results indicate that CSDS markedly affects NREM sleep, with detrimental effects leading to an insomniac-like pattern in mice. As insomnia is highly prevalent in mood disorders, our results further validate the CSDS paradigm to investigate the mechanisms of these pathologies.

Interestingly, we also found that only susceptible mice anticipated the stress session even after cessation of the CSDS paradigm. Five days after the end of the CSDS paradigm, we found an anticipatory arousal characterized by increment of wake at the time corresponding to the SD session (7–10 a.m.). A comparable trend was observed 3 weeks after cessation of the stress paradigm (data not shown). Anticipation of arousal is classically described under food entrainment in rodents (Mistlberger, 2011; Castro-Faúndez et al., 2016). It usually emerges after a few days of scheduled feeding and depends on food-entrainable oscillators. Our stress paradigm elicited similar temporal sequence as a result of the repeated stress schedule throughout the 10-day CSDS for the purpose of the sleep analysis. Interestingly, excessive anticipatory reactions to potential threat are a common feature across anxiety disorders (Grupe and Nitschke, 2013). Absence of such anticipatory response in unsusceptible mice might reveal physiological adaptations that contribute to coping mechanisms. Conversely, in susceptible mice, anticipatory wake along with long-lasting social aversion, insomniac-like profile, and anxiety-like behavior may offer a more complete picture of the detrimental consequences of chronic stress, paralleling those found in anxiety disorders.

To conclude, the results presented here show that temporal organization of vigilance states is disrupted throughout and following 10-day CSDS. We propose that sleep alterations occurring all along the course of CSDS might participate in the recovery processes to cope with stress or the processing of emotional memory without impeding negative stress outcomes. These long-term deleterious consequences of CSDS in the most vulnerable individuals include hyperarousal during NREM sleep reported in primary insomnia and stress-anticipated arousal found in anxious patients. The CSDS model thus provides significant sleep similarities with stress-related diseases in humans and position sleep as a major player regulating stress consequences.

## Authors contributions

FH, VV, SEM, and VF designed research; FH, VV, and VF performed the experiments and analyzed the data. All authors contributed to finalize the manuscript for submission for publication.

### Conflict of interest statement

The authors declare that the research was conducted in the absence of any commercial or financial relationships that could be construed as a potential conflict of interest.
