# Supplementary material for: Effects of Social Defeat Stress on Sleep in Mice
Source: Front Behav Neurosci. 2017 Nov 28;11:227. doi: 10.3389/fnbeh.2017.00227 (PMC5712311; doi:10.3389/fnbeh.2017.00227)
Supplement: Supplementary file 1 [file Table1.docx]

**Table S1.** Statistical analysis for figures 1-6 and tables 1-2.

Statistical tests for each figure/table and panel, with the test used (non-parametric analysis are indicated when used with the Friedman annotation for ANOVAs), the number of mice (n), the F/t value and multiple comparisons used when appropriate are indicated in the *post-hoc* test column.

|  |  |  |  |  |  |  |  |
| --- | --- | --- | --- | --- | --- | --- | --- |
| **Figure # Table #** | **Panel** | **Test used**  Alpha = 0.05 | **n** | **Factor** | **F value** | **P value** | ***Post-hoc* tests** |
| **1C** | Interaction  Time | 2-way RM ANOVA | 9 | Stress  Susceptibility  Interaction | F(1,14) = 22.96  F(1,14) = 0.802  F(1,14) = 36.74 | P=0.0003  P=0.3558  P<0.0001 | Sidak’s |
|  |  |  |  |  |  |  |  |
| **2** | Wake | 1-way RM ANOVA  FRIEDMAN  1-way RM ANOVA | 9 | Stress | **10-13h**  F(3,24) = 7.11  **13-16h**  **Dark period**  F(3,24) = 18.24 | **10-13h**  P=0.0014  **13-16h**  P=0.4346  **Dark period**  P<0,0001  P<0,0001 | Tukey’s  None  Tukey’s |
|  | NREM sleep | 1-way RM ANOVA  FRIEDMAN  1-way RM ANOVA | 9 | Stress | **10-13h**  F(3,24) = 4.356  **13-16h**  **Dark period**  F(3, 24) = 16.92 | **10-13h**  P=0.0138  **13-16h**  P=0.2688  **Dark period**  P<0.0001  P<0,0001 | Tukey’s  None  Tukey’s |
|  | REM sleep | 1-way RM ANOVA  FRIEDMAN | 9 | Stress | **10-13h**  F(3,24) = 18.64  **13-16h**  F(3,24) = 1.294  **Dark period** | **10-13h**  P<0.0001  **13-16h**  P=0.2992  **Dark period**  P=0.0008  P=0,0008 | Tukey’s  None  Dunn’s |
|  |  |  |  |  |  |  |  |
| **3B** | NREM sleep  10-13h | 1-way RM ANOVA  FRIEDMAN | 8 | Stress | Delta  Theta  F(3,21) = 18.31  Alpha  F(3,21) = 38.43  Beta  F(3,21) = 30.69  Gamma  F(3,21) = 24.10 | P=0.001  P<0.0001  P<0.0001  P<0.0001  P<0.0001 | Dunn’s  Tukey’s  Tukey’s  Tukey’s  Tukey’s |
| **3C** | NREM sleep  13-16h | 1-way RM ANOVA | 8 | Stress | Delta  F(3,21) = 9.358  Theta  F(3,21) = 8.660  Alpha  F(3,21) = 8.038  Beta  F(3,21) = 7.406  Gamma  F(3,21) = 9.809 | P=0.0115  P=0.0006  P=0.0009  P=0.0014  P=0.0003 | Tukey’s  Tukey’s  Tukey’s  Tukey’s  Tukey’s |
| **3D** | NREM sleep  Dark period | 1-way RM ANOVA | 8 | Stress | Delta  F(3,21) = 3.628  Theta  F(3,21) = 2.326  Alpha  F(3,21) = 5.125  Beta  F(3,21) = 5.698  Gamma  F(3,21) = 3.245 | P=0.0297  P=0.1039  P=0.0081  P=0.0279  P=0.0424 | Tukey’s: all ns  none  Tukey’s  Tukey’s  Tukey’s |
|  |  |  |  |  |  |  |  |
|  |  |  |  |  |  |  |  |
| **4B** | Wake  NREM  REM | 1-way RM ANOVA  FRIEDMAN for all | 7 | Stress |  | P=0.0012  P=0.0084  P=0.0005 | Dunn’s  Dunn’s  Dunn’s |
| **4C** | NREM sleep | 1-way RM ANOVA  FRIEDMAN for all | 7 | Stress |  | Delta  P=0.0003  Theta  P=0.1118  Alpha  P=0.0012  Beta  P=0.0027  Gamma  P=0.1118 | Dunn’s  none  Dunn’s  Dunn’s  none |
| **4D** | Wake  NREM  REM | 1-way RM ANOVA  FRIEDMAN for all | 7 | Stress |  | P=0.0027  P=0.0027  P=0.0027 | Dunn’s  Dunn’s  Dunn’s |
|  |  |  |  |  |  |  |  |
| **5A1** | Wake | 2-way RM ANOVA | 9 | Stress  Time  Interaction | F(1,8) = 5.589  F(7,56) = 61.8  F(7,56) = 2.274 | P=0.0457  P<0.0001  P=0.0412 | Sidak’s |
| **5A2** | NREM sleep | 2-way RM ANOVA | 9 | Stress  Time  Interaction | F(1,8) = 5.011  F(7,56) = 53.51  F(7,56) = 2.094 | P=0.0556  P<0.0001  P=0.0591 | none |
| **5A3** | REM sleep | 2-way RM ANOVA | 9 | Stress  Time  Interaction | F(1,8) = 0.9502  F(7,56) = 59.46  F(7,56) = 2.519 | P=0.3582  P<0.0001  P=0.0252 | Sidak’s |
| **5B1** | Wake | 2-way RM ANOVA | 7 | Stress  Time  Interaction | F(1,7) = 0.4876  F(7,42) = 94.92  F(7,42) = 1.438 | P=0.5111  P<0.0001  P=0.2161 | none |
| **5B2** | NREM sleep | 2-way RM ANOVA | 7 | Stress  Time  Interaction | F(1,7) = 2.558  F(7,42) = 64.64  F(7,42) = 0.6287 | P=0.1609  P<0.0001  P=0.7293 | none |
| **5B3** | REM sleep | 2-way RM ANOVA | 7 | Stress  Time  Interaction | F(1,7) = 1.057  F(7,42) = 36.33  F(7,42) = 1.752 | P=0.3436  P<0.0001  P=0.1230 | none |
|  |  |  |  |  |  |  |  |
|  |  |  |  |  |  |  |  |
| **6A1** | NREM sleep delta | Wilcoxon t test | 8 | Light  Dark |  | P=0.1953  P=0.0078 | Compared to BL |
|  | NREM sleep theta | paired t test  two-tailed | 8 | Light  Dark |  | P=0.2082  P=0.1396 | Compared to BL |
|  | NREM sleep alpha | Wilcoxon t test | 8 | Light  Dark |  | P=0.7422  P=0.0547 | Compared to BL |
|  | NREM sleep beta | paired t test  two-tailed | 8 | Light  Dark |  | P=0.0255  P=0.0177 | Compared to BL |
|  | NREM sleep gamma | Wilcoxon t test | 8 | Light  Dark |  | P=0.1953  P<0.0078 | Compared to BL |
| **6A2** | REM sleep delta | Wilcoxon t test | 8 | Light  Dark |  | P=0.6406  P=0.9453 | Compared to BL |
|  | REM sleep theta | Wilcoxon t test | 8 | Light  Dark |  | P=0.4609  P=0.2500 | Compared to BL |
|  | REM sleep alpha | paired t test  two-tailed | 8 | Light  Dark |  | P=0.0494  P=0.1857 | Compared to BL |
|  | REM sleep beta | Wilcoxon t test | 8 | Light  Dark |  | P>0.9999  P>0.9999 | Compared to BL |
|  | REM sleep gamma | paired t test  two-tailed | 8 | Light  Dark |  | P=0.3911  P=0.0341 | Compared to BL |
| **6B1** | NREM sleep delta | Wilcoxon t test | 7 | Light  Dark |  | P=0.0781  P=0.0156 | Compared to BL |
|  | NREM sleep theta | Wilcoxon t test | 7 | Light  Dark |  | P=0.0313  P=0.0156 | Compared to BL |
|  | NREM sleep alpha | Wilcoxon t test | 7 | Light  Dark |  | P=0.1563  P=0.0469 | Compared to BL |
|  | NREM sleep beta | Wilcoxon t test | 7 | Light  Dark |  | P=0.0781  P=0.0156 | Compared to BL |
|  | NREM sleep gamma | Wilcoxon t test | 7 | Light  Dark |  | P=0.0781  P=0.0781 | Compared to BL |
| **6B2** | REM sleep delta | Wilcoxon t test | 7 | Light  Dark |  | P>0,9999  P=0.6875 | Compared to BL |
|  | REM sleep theta | Wilcoxon t test | 7 | Light  Dark |  | P=0.0781  P=0.1563 | Compared to BL |
|  | REM sleep alpha | Wilcoxon t test | 7 | Light  Dark |  | P=0.9375  P=0.5781 | Compared to BL |
|  | REM sleep beta | Wilcoxon t test | 7 | Light  Dark |  | P=0.8125  P=0.4688 | Compared to BL |
|  | REM sleep gamma | Wilcoxon t test | 7 | Light  Dark |  | P=0.1563  P=0.1094 | Compared to BL |
|  |  |  |  |  |  |  |  |
| **Table 1** | Bout number  Wake | 1-way RM ANOVA  FRIEDMAN | 9 | Stress | **10-13h**  **13-16h**  F(3,24) = 2.27  **Dark**  F(3,24) = 5.215 | **10-13h**  P=0.1499  **13-16h**  P=0.1061  **Dark**  P=0.0065 | none  none  Tukey’s |
|  | Bout number  NREM sleep | 1-way RM ANOVA  FRIEDMAN | 9 | Stress | **10-13h**  **13-16h**  F(3,24) = 2.417  **Dark**  F(3,24) = 5.383 | **10-13h**  P=0.1240  **13-16h**  P=0.0911  **Dark**  P=0.0056 | none  none  Tukey’s |
|  | Bout number  REM sleep | 1-way RM ANOVA  FRIEDMAN | 9 | Stress | **10-13h**  **13-16h**  F(3,24) = 0.4859  **Dark**  F(3,24) = 9.742 | **10-13h**  P=0.0004  **13-16h**  P=0.6952  **Dark**  P=0.0002 | Dunn’s  none  Tukey’s |
|  | Bout mean duration  Wake | 1-way RM ANOVA  FRIEDMAN  FRIEDMAN | 9 | Stress | **10-13h**  **13-16h**  **Dark** | **10-13h**  P=0.0571  **13-16h**  P=0.1084  **Dark**  P=0.0010 | none  none  Dunn’s |
|  | Bout mean duration  NREM sleep | 1-way RM ANOVA  FRIEDMAN | 9 | Stress | **10-13h**  F(3,24) = 3.632  **13-16h**  F(3,24) = 0.9475  **Dark** | **10-13h**  P=0.0272  **13-16h**  P=0.4333  **Dark**  P=0.6149 | Tukey’s: all ns  none  none |
|  | Bout mean duration  REM sleep | 1-way RM ANOVA  FRIEDMAN | 9 | Stress | **10-13h**  **13-16h**  F(3,24) = 0.7792  **Dark**  F(3,24) = 0.3209 | **10-13h**  P=0.0302  **13-16h**  P=0.5172  **Dark**  P=0.8101 | Dunn’s : all ns  none  none |
|  |  |  |  |  |  |  |  |
| **Table 2** | Bout number  Wake | 1-way RM ANOVA  FRIEDMAN for all | 7 | Stress | **10-13h**  **Dark** | **10-13h**  P=0.1403  **Dark**  P=0.0003 | none  Dunn’s |
|  | Bout number  NREM sleep | 1-way RM ANOVA  FRIEDMAN for all | 7 | Stress | **10-13h**  **Dark** | **10-13h**  P=0.0718  **Dark**  P=0.0003 | none  Dunn’s |
|  | Bout number  REM sleep | 1-way RM ANOVA  FRIEDMAN for all | 7 | Stress | **10-13h**  **Dark** | **10-13h**  P=0.0002  **Dark**  P=0,0084 | Dunn’s  Dunn’s |
|  | Bout mean duration  Wake | 1-way RM ANOVA  FRIEDMAN for all | 7 | Stress | **10-13h**  **Dark** | **10-13h**  P=0.1118  **Dark**  P=0.0003 | none  none |
|  | Bout mean duration  NREM sleep | 1-way RM ANOVA  FRIEDMAN for all | 7 | Stress | **10-13h**  **Dark** | **10-13h**  P=0.0300  **Dark**  P=0.7682 | Dunn’s  none |
|  | Bout mean duration  REM sleep | 1-way RM ANOVA  FRIEDMAN for all | 7 | Stress | **10-13h**  **Dark** | **10-13h**  P=0.0352  **Dark**  P=0.9640 | Dunn’s  none |
| **Table 1** | Bout number  Wake | 1-way RM ANOVA  FRIEDMAN | 9 | Stress | **10-13h**  **13-16h**  F(3,24) = 2,27 | **10-13h**  P=0,1499  13-16h  P=0.1061 | none  none |
